# *Pennisetum purpureum* Schumach Supplementation Enhances Grip Strength in Adults with Low Muscle Mass: A Randomized Controlled Trial

**DOI:** 10.7150/ijms.124224

**Published:** 2026-02-04

**Authors:** Shih-Wei Huang, Ben-Mao Liu, Yu-Hao Lee, Megan F. Liu, Eisuke Ochi, Chii-Horng Wei, Ming-Ta Yang

**Affiliations:** 1 Department of Physical Medicine and Rehabilitation, Wan Fang Hospital, Taipei Medical University, Taipei, Taiwan.; 2 Department of Physical Medicine and Rehabilitation, School of Medicine, Taipei Medical University, Taipei, Taiwan.; 3 Department of Physical Medicine and Rehabilitation, Hsin Kuo Min Hospital, Taipei Medical University, Taoyuan, Taiwan.; 4 Department of Physical Medicine and Rehabilitation, Shuang Ho Hospital, Taipei Medical University, New Taipei City, Taiwan.; 5 Department of Orthopedic Surgery, Shuang Ho Hospital, Taipei Medical University, New Taipei City, Taiwan.; 6 School of Gerontology and Long-Term Care, College of Nursing, Taipei Medical University, Taipei, Taiwan.; 7 Faculty of Bioscience and Applied Chemistry, Hosei University, Tokyo, Japan.; 8 Graduate School of Sports and Health Studies, Hosei University, Tokyo, Japan.; 9 Center for General Education, Tungnan University, New Taipei City, Taiwan.; 10 Center for General Education, Taipei Medical University, Taipei, Taiwan.; 11 Clinical Research Center, Taipei Medical University Hospital, Taipei, Taiwan.

**Keywords:** antioxidant supplementation, grip strength, liver and kidney function, physical performance, testosterone

## Abstract

**Background:**

Low muscle mass and reduced muscle strength are prevalent among adults, particularly in the aging population, and are associated with an increased risk of sarcopenia-related complications. *Pennisetum purpureum* Schumach is a plant known for its antioxidant properties, but its effects on muscle strength and function in adults with low muscle mass remain unexplored. In this study, we investigated the effects of *Pennisetum purpureum Schumach* supplementation on liver and kidney function, hormonal markers, anthropometric measurements, and physical performance in adults with low muscle mass.

**Methods:**

A 12-week randomized, double-blind, placebo-controlled trial was conducted with 35 participants with low muscle mass and reduced muscle strength. The experimental group received 300 mg of *Pennisetum purpureum* Schumach extract three times daily, while the placebo group received a placebo. Assessments included grip strength, muscle mass, liver and kidney function, and a 10-meter walk test at baseline, 8 weeks, and 12 weeks.

**Results:**

The experimental group showed a significant increase in grip strength (*p* < 0.05) after 8 and 12 weeks compared to baseline. No significant changes in muscle mass or performance in the 10-meter walk test were observed. Liver and kidney function remained unaffected in both groups.

**Conclusion:**

*Pennisetum purpureum* Schumach supplementation was associated with an increase in grip strength after 8 and 12 weeks. No definitive effects were identified for muscle mass, walking performance, liver or kidney function, or hormonal markers. Observationally, some variability in testosterone levels was noted among male participants; however, the study lacked the design and statistical power necessary to determine hormonal effects. These findings suggest that the supplementation may be safe and potentially useful, although larger and parameter-comprehensive trials are required to verify physiological mechanisms and confirm efficacy.

## Introduction

Sarcopenia, characterized by the progressive loss of muscle mass, strength, and physical performance, is a common skeletal muscle disorder that affects approximately 10-16% of the global elderly population. It is associated with numerous adverse outcomes, including an increased risk of falls, frailty, and mortality 1. Although sarcopenia is more prevalent in older adults, the decline in muscle mass typically begins around the age of 40 2-3. Consequently, its detrimental effects on quality of life, healthcare utilization, morbidity, and mortality can affect both middle-aged and older adults 1-2, 4. In addition to aging, factors such as being underweight, female sex, and chronic disease further increase the risk of sarcopenia and its associated health consequences 5.

The pathophysiology of sarcopenia is multifactorial, involving biological alterations in muscle structure, hormonal imbalances, inflammation, and external factors such as inadequate energy intake 6. Effective management strategies require a comprehensive understanding of these mechanisms and should integrate both non-pharmacological and pharmacological approaches. Currently, non-pharmacological interventions remain the mainstay of treatment, as there are no FDA-approved pharmacotherapies specifically for sarcopenia. Resistance training is widely recognized as the cornerstone of sarcopenia management, supported by substantial evidence of its efficacy 7-8. From a nutritional perspective, interventions such as adequate protein intake, vitamin D supplementation, and antioxidant-rich nutrients have shown potential benefits in mitigating muscle loss and improving function in some studies 9.

*Pennisetum purpureum* Schumach, commonly known as elephant grass or napiergrass, is widely cultivated in Taiwan, where it is primarily used as livestock feed. In recent years, napiergrass has also been incorporated into health-promoting beverages; however, its potential health benefits have not been scientifically validated. Its antioxidative properties have been demonstrated through chemical analyses 10, and previous studies have identified it as a rich source of antioxidant compounds, underscoring its potential as a valuable natural source of phytochemicals 11. To date, no studies have evaluated the efficacy of *Pennisetum purpureum* Schumach supplementation in managing sarcopenia or related muscle decline. Given its established antioxidant properties, this study aimed to investigate its potential to enhance muscle strength and physical function. While diagnostic criteria for sarcopenia were referenced, the present study focused on adults exhibiting early signs of muscle decline, such as low muscle mass or reduced grip strength.

## Materials and Methods

### Participants

Participants were enrolled between June 2023 and December 2023 from our hospital and the surrounding community. Eligibility screening was conducted using bioelectrical impedance analysis (BIA) (InBody Home H20B). Individuals with skeletal muscle index (SMI) below 7.0 kg/m² for males or 5.7 kg/m² for females (assessed by BIA) were further assessed for inclusion. While the 2019 Asian Working Group for Sarcopenia (AWGS) criteria were referenced, the study focused on individuals exhibiting features of early-stage sarcopenia, including low muscle mass and reduced muscle strength 12. Inclusion criteria targeted individuals aged 18 to 75 exhibiting early signs of muscle decline, defined as whole-body DXA SMI < 7.0 kg/m² in men or < 5.4 kg/m² in women, grip strength < 28 kg in men or < 18 kg in women, or 5 times sit-to-stand and stand-to-sit taking more than 12 seconds in both genders. Meeting all inclusion criteria was necessary for study enrollment. Exclusion criteria included chronic cerebrovascular, cardiovascular, hepatic, renal, or diabetic mellitus diseases, participation in other research within the past 3 months, daily alcohol consumption habits, and use of other nutritional supplements during the study. Random allocation to either the *Pennisetum purpureum* Schumach extract supplement group or the placebo group was achieved by a research assistant using computer-generated random numbers and sealed envelopes. Baseline variables (age, sex, body height, body weight, and BMI) were recorded upon enrollment, and the research assistant, upon revealing the patient's group allocation, prepared the intervention. A blinded research member provided the intervention, and outcome measures were analyzed by an assessor who remained unaware of group allocations. Patients provided written informed consent after receiving an explanation of the study's aim and procedures. Three participants dropped out of the study due to refused treatment, but no serious adverse events were reported throughout the study. Therefore, 35 participants were included in the final analysis.

### Experimental design

**Figure 1:**
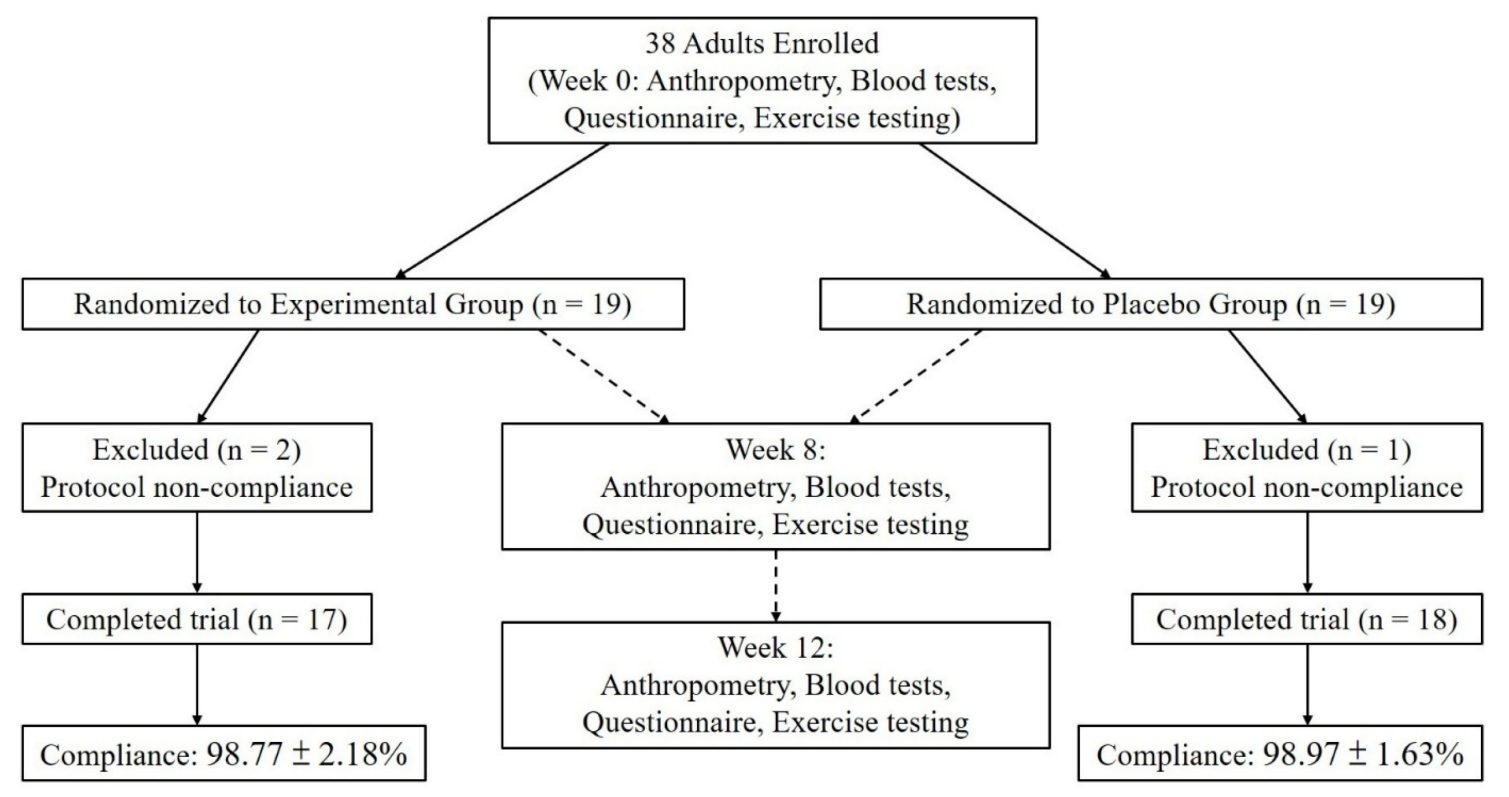
Participant flow chart showing enrollment, randomization, follow-up, and completion of the trial.

This single-center, randomized, double-blind, placebo-controlled trial was conducted at a medical university hospital, with 35 participants randomly assigned to an experimental group (n = 17) and a placebo group (n = 18). When comparing baseline demographic variables—including age, sex, body weight, and BMI—no statistically significant differences were observed between the two groups, except for body height, which showed a statistically significant difference (*p* < 0.05;** Table 1**). Participants recorded their dietary intake using a 3-day food log (two weekdays and one weekend day, on non-consecutive days) at baseline, week 8, and week 12 during the supplementation period with *Pennisetum purpureum* Schumach. The results indicated no significant differences in the intake of energy, carbohydrates, protein, or fat between the two groups before, during, or after supplementation. Thus, dietary intake was deemed consistent across participants throughout the intervention period (**Table 2**). The study adhered to the principles of the Declaration of Helsinki and was approved by the Institutional Review Board (IRB) of Taipei Medical University (IRB no. N202305092). Additionally, the trial was registered at ClinicalTrials.gov (NCT05911516). The flow of participants through the study is shown in** Fig. 1**.

### Supplementation

The *Pennisetum purpureum* Schumach extract and placebo were produced by Natural Keeper Enterprise Co., Ltd. The *Pennisetum purpureum* Schumach alopecuroides dried grass used in this study was sourced from Hualien County, Taiwan. The raw material was extracted using water at a 1:14.6 (w/w) ratio at 95-100 °C for 1 hour. The resulting extracts were filtered, and the remaining residue was re-extracted under the same conditions one additional time. The combined filtrates were concentrated and freeze-dried to produce a dry powder. This dry powder was mixed with excipients including maltodextrin, magnesium octadecenoate, and silicon dioxide. Each capsule contained 300 mg of the active powder (API). The placebo capsules contained only maltodextrin, magnesium octadecenoate, and silicon dioxide, with a total content of approximately 200 mg. Throughout the 12-week supplementation phase of this experiment, participants in the *Pennisetum purpureum* Schumach extract group consumed one capsule three times per day. The placebo capsules were visually indistinguishable from the *Pennisetum purpureum* Schumach extract capsules. Compliance was evaluated by counting the number of remaining capsules.

### Outcome assessment

At weeks 8 and 12 of the intervention, various assessments were performed by a trained research assistant who was blinded to the participants' group allocation. These assessments included anthropometric measurements, body composition analysis, blood pressure monitoring, hormone profiling, liver and renal function tests, physical performance evaluations, and collection of a 3-day dietary record.

Anthropometric measurements included mid-upper arm circumference (MUAC) and mid-thigh circumference (MTC). Whole-body dual-energy X-ray absorptiometry (DXA; Hologic HORIZON-W, MA, USA) was used to assess appendicular skeletal muscle mass index (ASMI). Hormonal analyses measured testosterone, cortisol, insulin-like growth factor 1 (IGF-1), growth hormone, follistatin, and myostatin. Liver function was assessed by measuring glutamic oxaloacetic transaminase (GOT; also known as aspartate aminotransferase, AST) and glutamic pyruvic transaminase (GPT; also known as alanine aminotransferase, ALT), while renal function was evaluated through blood urea nitrogen (BUN) and creatinine (CRE) levels. According to Kalas et al. (2021), serum alanine aminotransferase (ALT) and aspartate aminotransferase (AST) are the primary and most reliable indicators of hepatocellular injury. A disproportionate increase in ALT and AST levels reflects hepatocellular damage and serves as the clinical basis for assessing liver safety in both diagnostic and interventional settings. Therefore, the assessment of liver function using GOT and GPT is widely recognized as the standard and effective approach for evaluating hepatic safety in human clinical trials 13.

Physical function was assessed using grip strength and the 10-meter walking test. Grip strength was measured with a hand dynamometer (Baseline 12-0240) using the dominant hand, with the elbow flexed at a 90° angle and the arm held close to the body. The test was performed three times, and the highest value was recorded. In the 10-meter walking test, participants walked as quickly as possible over a 10-meter distance. Time was recorded with a stopwatch, and walking speed (m/s) was calculated by dividing the distance by time. This test was repeated three times, and the best result was recorded. A 3-day dietary record (two weekdays and one weekend day on non-consecutive days) was collected and analyzed for total energy, carbohydrate, protein, and fat intake.

### Statistical analysis

We used G*Power (version 3.1.9.2, UCLA, Los Angeles, CA) to perform preliminary power analysis using the independent t-test and compared between-group differences in grip strength (2.9 kg, MCID) between baseline and 12 weeks after intervention 14. The result suggested that at least 31 participants were needed to achieve the appropriate power ([1-β] = .85; α = 0.05). Descriptive statistics for study variables were presented separately for each study group. For normally distributed continuous variables, mean and standard deviation values were provided, while for non-normally distributed variables, median and interquartile range values were reported. Categorical variables were summarized using frequencies and percentages. Statistical testing was conducted at a significance level of 0.05, and confidence intervals (CIs) were calculated at a confidence level of 95%. Demographic and baseline characteristics were summarized and compared between study groups using one-way analysis of variance (ANOVA) for continuous variables and Fisher's exact test for categorical variables. Group differences in normally distributed efficacy outcomes, adjusted for sex, were assessed using analysis of covariance. Post-intervention data analyses were adjusted for baseline measurements. Group differences were examined using the Kruskal-Wallis test for non-normally distributed data and Fisher's exact test for nominal categorical outcomes. Changes between baseline and post-intervention outcomes within each study group were assessed using the paired t-test for normally distributed outcomes, the Wilcoxon signed-rank test for non-normally distributed outcomes, and the McNemar test for binary outcomes. Normality of the data was assessed using the Shapiro-Wilk test, and homogeneity of variance was assessed using Levene's test. The analyses of this study were performed using SPSS (version 22.0). Statistical significance was set at *p* < 0.05.

## Results

### Effects of *Pennisetum purpureum* Schumach extract supplementation on liver and kidney function

The changes in liver and kidney function before, during, and after supplementation with *Pennisetum purpureum* Schumach for 12 weeks are shown in **Table 3**. The results indicate that there were no significant differences in liver function (GOT, GPT) and kidney function (BUN, CRE) indicators between the two groups of experimental participants before, during, and after supplementation. Overall, no apparent adverse changes in liver or kidney parameters were observed during the 12-week supplementation period.

### Effects of *Pennisetum purpureum* Schumach extract supplementation on hormonal profiles

The effects of 12 weeks of supplementation with *Pennisetum purpureum* Schumach on the hormonal profiles before, during, and after supplementation, as shown in** Table 4**, indicated no significant differences in the concentrations of testosterone, cortisol, IGF-1, growth hormone, follistatin, and myostatin between the two groups of participants throughout the supplementation period, as assessed by mixed-design two-way ANOVA. Furthermore, although independent t-tests revealed that the experimental group had significantly higher testosterone concentrations after 8 and 12 weeks of supplementation compared to the placebo group (*p* < 0.05), the significant difference might be due to the large disparity in the number of male participants between the experimental group (8 males) and the placebo group (4 males). Further analysis comparing the changes in testosterone concentrations between male and female participants in both groups showed no significant differences, indicating that the *Pennisetum purpureum* Schumach supplementation did not effectively increase testosterone concentrations. However, upon further examination of the raw data using the percentage of changes, it was found that female participants in both the experimental and placebo groups showed no significant changes in testosterone concentration. In contrast, among the 8 male participants in the experimental group, 5 showed an increase in testosterone concentration of over 5% after 12 weeks of supplementation, with 2 even showing an increase of over 30%. Conversely, male participants in the placebo group showed no significant changes. Therefore, slight fluctuations in testosterone concentrations were observed among some male participants, but the current study cannot determine their physiological relevance.

### Effects of *Pennisetum purpureum* Schumach extract supplementation on anthropometric measurements

The changes in anthropometric measurements before, after 8 weeks and after 12 weeks of supplementation with *Pennisetum purpureum* Schumach are shown in **Table 5**. The results indicate that there were no significant differences between the two groups in BMI, blood pressure (systolic and diastolic), pulse rate, SMI, and upper arm circumference before, during, and after 12 weeks of supplementation. Additionally, analysis of variance with mixed-design two-way ANOVA revealed no interaction for body mass index and thigh circumference, while the main effect of time showed that all participants significantly increased their body mass index and thigh circumference after 8 and 12 weeks of supplementation compared to before supplementation. In other words, all participants in the study showed an increase in body mass index and thigh circumference during the supplementation period, but this phenomenon was not attributed to the benefits of intervention.

### Effects of *Pennisetum purpureum* Schumach extract supplementation on physical function

The physical function before, after 8 weeks, and after 12 weeks of supplementation are presented in **Table 6**. The ANOVA revealed a significant interaction effect between the groups and time for grip strength (*p* < 0.05). Further analysis of the main effects revealed that the experimental group showed a significant increase in grip strength after 8 and 12 weeks of supplementation compared to baseline. However, there were no significant differences in grip strength for the placebo group before and after supplementation, and there were no significant differences between the two groups. Additionally, there were no significant differences between the two groups in the 10-meter walk test before and after supplementation. These results indicate that continuous supplementation of *Pennisetum purpureum* Schumach at a daily dose of 3 times 300 mg for 12 weeks has a positive effect on grip strength but does not improve performance in the 10-meter walk test.

## Discussion

In a 12-week double-blinded randomized placebo-controlled trial, supplementation with *Pennisetum purpureum* Schumach was associated with improved grip strength, whereas the liver and kidney function markers measured in this study showed no apparent changes. Despite this improvement, no significant changes were observed in hormone levels, body composition, or other secondary parameters. Further analysis, however, revealed an upward trend in testosterone levels among male participants with low muscle mass. These findings suggest that *Pennisetum purpureum* Schumach may serve as a safe, plant-derived option for enhancing muscle strength in individuals with early signs of muscle decline.

To better understand the potential effects of *Pennisetum purpureum* Schumach supplementation on age-related muscle deterioration, it is essential to consider the underlying pathophysiology of muscle loss. Aging disrupts skeletal muscle homeostasis, resulting in an imbalance between anabolic and catabolic signaling pathways that regulate protein metabolism. These alterations are accompanied by reductions in both the size and number of type II muscle fibers, as well as increased infiltration of intramuscular and intermuscular fat. In addition, aging contributes to a decline in satellite cell populations, which are critical for muscle regeneration and repair 15-16. Satellite cell function may also be impaired due to changes in systemic regulatory factors affecting their proliferation and differentiation. Several additional mechanisms contribute to progressive muscle loss, including neuromuscular junction dysfunction, a decline in motor unit numbers, chronic low-grade inflammation, insulin resistance, and increased oxidative stress 17-19. Denervation and the subsequent replacement of muscle tissue with adipose tissue further exacerbate muscle mass loss and functional decline.

We hypothesized that the antioxidant properties of *Pennisetum purpureum* Schumach supplementation may contribute to improvements in muscle function, particularly in individuals with early signs of muscle decline. Prior studies have demonstrated that this plant is rich in minerals, vitamins, and a variety of antioxidant compounds 20. The red pigmentation of its stems and leaves, attributed to the presence of anthocyanins, has been proposed as an indicator of its antioxidant capacity 11. Polyphenols have been identified as the major contributors to its antioxidant activity 10, 19. Furthermore, aqueous extracts of *Pennisetum purpureum* Schumach have been shown to reduce oxidative damage to biomolecules and enhance the activity of endogenous antioxidant enzymes. These extracts exhibited strong free radical scavenging ability, reducing power, and ferrous ion chelation capacity 21. To comprehensively evaluate the physiological adaptations, key anabolic and catabolic hormones (testosterone, cortisol, IGF-1, growth hormone, follistatin, and myostatin) were measured, as these biomarkers collectively reflect the endocrine regulation of muscle strength and protein metabolism. Given the study's focus on muscle-related anabolic responses rather than gonadal axis regulation, key hormones directly involved in muscle protein metabolism were prioritized. Future studies with larger sample sizes and extended sampling protocols should include these additional sex hormones and SHBG to clarify the full spectrum of endocrine responses and to determine whether *Pennisetum purpureum* Schumach exerts sex-specific hormonal modulation.

Our investigation revealed that supplementation with *Pennisetum purpureum* Schumach may be associated with a slight upward trend in testosterone levels among male participants with low muscle mass. Testosterone has been implicated in the maintenance of muscle strength and function through multiple mechanisms, including promoting the differentiation of mesenchymal stem cells into muscle rather than adipose tissue, stimulating satellite cell proliferation, enhancing muscle protein synthesis, and inhibiting protein degradation 22. These hormonal markers were selected to reflect the anabolic-catabolic balance and endocrine regulation associated with muscle strength and metabolic adaptation. Previous clinical studies have reported significant associations between low testosterone levels and reduced handgrip strength, such as in male kidney transplant recipients 23, and among community-dwelling older men 24. Moreover, testosterone replacement therapy has been consistently shown to increase lean body mass in elderly men 25. In the present study, although no significant changes in muscle mass were observed, grip strength improved following supplementation. This observation aligns with previous findings that muscle strength tends to decline earlier and more rapidly than muscle mass during age-related muscle loss 1. This underscores the need to consider muscle mass and muscle function as distinct, though related, components. Supporting this distinction, Van den Beld et al. reported that serum testosterone levels were associated with muscle strength but not with muscle mass in healthy, independently living older men 26.

The improvement in grip strength observed in this study may be partly due to the antioxidant properties of *Pennisetum purpureum* Schumach, which could influence endocrine function and muscle contractility. Oxidative stress is known to impair skeletal muscle performance by disrupting mitochondrial function, accelerating protein degradation, and suppressing anabolic signaling 27. Bioactive compounds in *Pennisetum purpureum* Schumach, including polyphenols and anthocyanins, may counteract oxidative stress by scavenging free radicals and improving redox homeostasis 28-29, potentially helping to preserve testosterone levels, as oxidative stress can impair Leydig cell steroidogenesis 30. This sequential pathway, from antioxidant defense to hormonal modulation and subsequently enhanced neuromuscular performance, provides a plausible explanation for the observed findings. However, as our study did not directly measure oxidative stress biomarkers, mitochondrial function, or neuromuscular activation, this proposed mechanism remains speculative and warrants further investigation.

Several limitations of this study should be acknowledged. First, participants were not exclusively older adults with clinically diagnosed sarcopenia, but rather individuals with low muscle mass or early indicators of muscle decline. Although this may limit the direct generalizability of the findings to populations with clinically diagnosed sarcopenia, the results provide broader insights that could inform future subgroup analyses specifically targeting elderly individuals with confirmed sarcopenia. Second, although the sample size was calculated based on the minimal clinically important difference (MCID) for grip strength in randomized controlled trials, it may have been insufficient to detect changes in other outcomes such as body composition or hormone levels. Moreover, although randomization was applied, the sex distribution between groups was not completely balanced, with a higher proportion of women in the placebo group. However, all efficacy analyses were adjusted for sex to minimize potential confounding effects, and no significant baseline differences were observed between groups. Future studies with larger and sex-stratified samples are warranted to provide a more comprehensive understanding of the efficacy of *Pennisetum purpureum* Schumach supplementation across a wider range of physiological outcomes. Finally, the potential influence of repeated functional assessments—such as grip strength and walking tests—on performance (i.e., training effects) cannot be entirely ruled out.

## Conclusions

This study investigated the effects of *Pennisetum purpureum* Schumach supplementation on muscle strength and mass in adults with low muscle mass or early signs of muscle decline. Participants who received the 12-week supplementation exhibited an improvement in grip strength, whereas the liver (GOT, GPT) and kidney (BUN, CRE) function markers measured in this study showed no apparent changes. However, no significant changes were observed in muscle mass, body composition, or physical function as measured by the 10-meter walk test.

These results indicate that an improvement in grip strength was observed in the supplementation group, although the underlying mechanisms remain to be elucidated. Future studies should investigate the supplement's synergistic effects when combined with resistance training and explore its efficacy in larger and more diverse populations, including older adults and individuals with chronic conditions. Additionally, examining the effects of extended supplementation durations and incorporating lifestyle factors such as diet and physical activity may offer further insights for optimizing strategies to prevent or manage age-related muscle decline.

## Figures and Tables

**Table 1:** Demographic variables of sarcopenia participants

Variable	Experimental group (n = 17)	Placebo group (n = 18)
Sex (M/F)	8/9	4/14
Age (years)	35.1 ± 17.7	45.3 ± 24.1
Height (cm)	164.0 ± 10.8 ^*^	156.8 ± 7.7
Weight (kg)	48.9 ± 5.4	46.2 ± 9.9
BMI (kg/m^2^)	18.3 ± 2.4	18.7 ± 3.2

Data are presented as the mean ± SD; ^*^Significant (*p* < 0.05) differences from placebo group; Experimental group: n = 17; Placebo group: n = 18.

**Table 2:** Dietary intake of participants during the experimental period

Variable	Week-0	Week-8	Week-12
Carbohydrate (g)			
Experimental group	175.91 ± 37.09	158.37 ± 46.36	170.93 ± 36.72
Placebo group	155.55 ± 37.93	157.59 ± 31.23	153.70 ± 38.07
Protein (g)			
Experimental group	55.65 ± 14.57	50.69 ± 11.33	51.28 ± 8.58
Placebo group	50.59 ± 15.52	50.55 ± 10.42	49.70 ± 9.98
Fat (g)			
Experimental group	59.86 ± 16.98	54.92 ± 13.90	55.13 ± 11.54
Placebo group	54.49 ± 16.24	52.40 ± 10.33	52.02 ± 10.97
Calories (kcal)			
Experimental group	1478.70 ± 324.72	1332.49 ± 316.17	1385.09 ± 261.34
Placebo group	1315.00 ± 288.78	1304.10 ± 211.59	1281.77 ± 253.09

Data are presented as the mean ± SD; Experimental group: n = 17; Placebo group: n = 18.

**Table 3:** Liver and kidney function of participants during the experiment period

Variable	Week-0	Week-8	Week-12
GOT (U/L)			
Experimental group	20.41 ± 6.12	20.77 ± 8.86	18.29 ± 4.67
Placebo group	23.67 ± 8.93	20.50 ± 5.54	22.56 ± 16.14
GPT (U/L)			
Experimental group	16.24 ± 7.47	19.88 ± 14.27	15.35 ± 6.08
Placebo group	17.92 ± 8.00	15.44 ± 5.90	14.58 ± 8.04
BUN (mg/dL)			
Experimental group	11.94 ± 3.53	12.35 ± 3.20	11.77 ± 2.61
Placebo group	12.28 ± 4.61	13.72 ± 4.07	13.00 ± 3.43
Creatinine (mg/dL)			
Experimental group	0.79 ± 0.17	0.81 ± 0.18	0.75 ± 0.19
Placebo group	0.73 ± 0.12	0.71 ± 0.15	0.71 ± 0.16

Data are presented as the mean ± SD; GOT: Glutamic oxaloacetic transaminase; GPT: Glutamic pyruvic transaminase; BUN: blood urea nitrogen; Experimental group: n = 17; Placebo group: n = 18.

**Table 4:** Hormonal profiles of participants during the experimental period

Variable	Week-0	Week-8	Week-12
Testosterone (nmol/L)			
Experimental group	2.91 ± 3.22	3.36 ± 3.92	3.27 ± 3.69
Placebo group	1.17 ± 2.19	0.99 ± 1.72	1.11 ± 2.05
Cortisol (µg/dL)			
Experimental group	8.86 ± 1.87	10.37 ± 3.17	10.05 ± 3.17
Placebo group	9.36 ± 3.05	11.71 ± 7.35	9.87 ± 8.16
IGF-1 (ng/mL)			
Experimental group	150.33 ± 49.17	141.98 ± 49.37	151.34 ± 50.17
Placebo group	156.68 ± 70.74	150.38 ± 61.45	146.57 ± 63.70
Growth hormone (ng/mL)			
Experimental group	0.95 ± 1.65	0.43 ± 0.67	0.50 ± 0.69
Placebo group	1.69 ± 2.58	1.18 ± 1.83	2.12 ± 3.59
Follistatin (ng/mL)			
Experimental group	2.29 ± 2.31	2.60 ± 2.96	2.90 ± 3.25
Placebo group	2.98 ± 3.46	2.78 ± 3.05	2.31 ± 2.42
Myostatin (ng/mL)			
Experimental group	13.04 ± 1.77	12.28 ± 2.69	12.77 ± 2.97
Placebo group	13.00 ± 3.44	13.86 ± 3.03	11.92 ± 2.10

Data are presented as the mean ± SD; IGF-1: Insulin-like growth factor-1; Experimental group: n = 17; Placebo group: n = 18.

**Table 5:** Anthropometric measurements of participants during the experimental period

Variable	Week-0	Week-8	Week-12
Body Mass Index (kg/m^2^)			
Experimental group	18.31 ± 2.42	18.66 ± 2.62^#^	18.63 ± 2.61^#^
Placebo group	18.74 ± 3.25	18.89 ± 3.13^#^	19.00 ± 3.30^#^
SBP (mmHg)			
Experimental group	111.29 ± 10.48	104.12 ± 10.37	109.47 ± 13.25
Placebo group	117.00 ± 18.04	113.22 ± 22.23	113.56 ± 17.99
DBP (mmHg)			
Experimental group	63.82 ± 8.10	59.88 ± 6.05	63.35 ± 10.34
Placebo group	67.94 ± 15.07	63.33 ± 8.04	64.17 ± 7.16
Pulse (bpm)			
Experimental group	83.53 ± 14.28	75.18 ± 9.02	71.65 ± 9.97
Placebo group	82.17 ± 14.16	74.17 ± 10.23	77.56 ± 9.91
ASMI (kg/m^2^)			
Experimental group	5.14 ± 0.67	5.13 ± 0.65	5.07 ± 0.66
Placebo group	4.87 ± 0.83	4.88 ± 0.81	4.85 ± 0.86
MUAC (cm)			
Experimental group	22.03 ± 2.16	22.12 ± 2.16	21.98 ± 2.10
Placebo group	21.64 ± 2.84	21.33 ± 2.74	21.98 ± 4.26
MTC (cm)			
Experimental group	38.85 ± 3.96	41.23 ± 4.06^#^	41.44 ±4.26^#^
Placebo group	38.94 ± 2.97	39.63 ± 3.18^#^	40.58 ± 2.86^#^

Data are presented as the mean ± SD; SBP, systolic blood pressure; DBP, diastolic blood pressure; ASMI, appendicular skeletal muscle index; MUAC, mid-upper arm circumference; MTC, mid-thigh circumference; ^#^ Significant (*p* < 0.05) differences from Week-0 for time main effect; Experimental group: n = 17; Placebo group: n = 18.

**Table 6:** Physical function of participants during the experimental period

Variable	Week-0	Week-8	Week-12
Hand grip strength (kg)			
Experimental group	18.74 ± 5.61	22.48 ± 5.61^*^	23.51 ± 6.38^*^
Placebo group	18.11 ± 6.03	19.35 ± 5.84	20.02 ± 5.93
10-m walk test (s)			
Experimental group	4.13 ± 0.71	4.22 ± 0.67	4.00 ± 0.52
Placebo group	5.02 ± 0.92	4.98 ± 0.72	4.92 ± 0.76

Data are presented as the mean ± SD; ^*^Significant (*p* < 0.05) differences from Week-0; Experimental group: n = 17; Placebo group: n = 18.

## Data Availability

The data supporting the findings of this study are available from the corresponding author upon reasonable request.
